# Median Preoptic Nucleus Mediates the Cardiovascular Recovery Induced by Hypertonic Saline in Hemorrhagic Shock

**DOI:** 10.1155/2014/496121

**Published:** 2014-11-18

**Authors:** Nathalia Oda Amaral, Lara Marques Naves, Marcos Luiz Ferreira-Neto, André Henrique Freiria-Oliveira, Eduardo Colombari, Daniel Alves Rosa, Angela Adamski da Silva Reis, Danielle Ianzer, Carlos Henrique Xavier, Gustavo Rodrigues Pedrino

**Affiliations:** ^1^Center for Neuroscience and Cardiovascular Physiology, Federal University of Goiás, Estrada do Campus s/n, 74690-900 Goiânia, GO, Brazil; ^2^Faculty of Physical Education, Federal University of Uberlândia, Rua Bejamin Constant 1286, 14801-903 Uberlândia, MG, Brazil; ^3^Department of Physiology and Pathology, São Paulo State University (UNESP), Rua Humaita 1680, 14801-903 Araraquara, SP, Brazil; ^4^Department of Biochemistry and Molecular Biology, Biological Sciences Institute, Federal University of Goiás, Estrada do Campus s/n, 74690-900 Goiânia, GO, Brazil; ^5^Department of Physiological Science, Universidade Federal de Goiás, Estrada do Campus s/n, P.O. Box 131, 74001-970 Goiânia, GO, Brazil

## Abstract

Changes in plasma osmolarity, through central and peripheral osmoreceptors, activate the median preoptic nucleus (MnPO) that modulates autonomic and neuroendocrine adjustments. The present study sought to determine the participation of MnPO in the cardiovascular recovery induced by hypertonic saline infusion (HSI) in rats submitted to hemorrhagic shock. The recordings of mean arterial pressure (MAP) and renal vascular conductance (RVC) were carried out on male Wistar rats (250–300 g). Hemorrhagic shock was induced by blood withdrawal over 20 min until the MAP values of approximately 60 mmHg were attained. The nanoinjection (100 nL) of GABA_A_ agonist (Muscimol 4 mM; experimental group (EXP)) or isotonic saline (NaCl 150 mM; control (CONT)) into MnPO was performed 2 min prior to intravenous overload of sodium through HSI (3 M NaCl, 1.8 mL/kg, b.wt.). Hemorrhagic shock reduced the MAP in control (62 ± 1.1 mmHg) and EXP (61 ± 0.4 mmHg) equipotently. The inhibition of MnPO impaired MAP (CONT: 104 ± 4.2 versus EXP: 60 ± 6.2 mmHg) and RVC (CONT: 6.4 ± 11.4 versus EXP: -53.5 ± 10.0) recovery 10 min after HSI. The overall results in this study demonstrated, for the first time, that the MnPO plays an essential role in the HSI induced resuscitation during hypovolemic hemorrhagic shock.

## 1. Introduction

Hemorrhagic shock remains a leading cause of death in trauma patients. Several cases of uncontrolled bleeding and its complications have resulted in the deaths of approximately 40% of these patients [1, 2]. In the last decades, series of studies have shown that hypertonic saline infusion (HSI) is beneficial to the prevention of hemorrhagic shock induced hypotension [3, 4]. In the 1980s, important study revealed that HSI was able to quickly restore blood pressure and cardiac output [5]. Although these hemodynamic responses to HSI in animals with hemorrhagic shock are already well established in the literature [6–9], there is still dearth of information about the involvement of central nervous system (CNS) in HSI induced resuscitation.

Recent studies in our laboratory have shown that selective denervation of carotid baroreceptors [10] or inactivation of the carotid chemoreceptors [11] abolished the recovery of blood pressure induced by HSI in hemorrhagic rats. This evidence corroborates the hypothesis that the HSI could activate neural reflex mechanisms for restoration of homeostasis. However, the regions of CNS that are involved in this recovery remain largely unknown.

Several studies have demonstrated the involvement of CNS's regions in the cardiovascular control and hydroelectrolytic balance in normovolemic rats. The noradrenergic neurons (A2 group) of the nucleus tractus solitarius (NTS) that receives projections from carotid afferents and peripheral osmoreceptors have been highlighted among these regions [12, 13]. In order to ensure specific cardiovascular responses, these inputs are transmitted to the median preoptic nucleus (MnPO) [14, 15]. The MnPO has been the focus of several studies as it regulates hydroelectrolytic balance and plays an important role in the cardiovascular homeostasis and body fluids control [16–21]. This is supported by the substantial activation of this region after an increase in osmolarity as unraveled by studies that involved the immediate activation of gene expression [22]. In agreement with these results, recent studies have identified that lesion of A1 and A2 neurons or blockade of adrenergic neurotransmission in MnPO abolishes the cardiovascular, autonomic, and endocrine responses induced by changes in volume or composition of body fluid homeostasis [13, 16, 17, 19, 23, 24].

Although MnPO is involved in the control of autonomic responses induced by HSI in normovolemic rats, no study has attempted to evaluate whether this region plays a role in the cardiovascular recovery induced by HSI in animals with hemorrhagic shock. Thus, we hypothesized that the information generated by changes in the composition of circulating blood volume from carotid sensors could relay in MnPO which in turn mediates necessary adjustments of autonomic and neuroendocrine systems for cardiovascular recovery. Hence, the aim of the present study was to investigate the effects of MnPO inhibition on cardiovascular recovery induced by HSI in hemorrhagic rats.

## 2. Methods

### 2.1. Animals

Adult male Wistar rats (250–300 g) were obtained from the central animal house of the* Universidade Federal de Goiás*. All experimental procedures were in strict adherence to Guidelines for Care and Use of Laboratory Animals as approved by the Ethics Committee of the Federal University of Goiás (protocol number 034/12).

### 2.2. Surgical Procedures

Rats were anesthetized with sodium thiopental (40 mg/kg, i.v.; Sigma-Aldrich, St. Louis, MO, USA) after induction with halothane (2% in 100% oxygen; Tanohalo; Cristália, Itapira, SP, Brasil). The right femoral artery was cannulated to record mean arterial pressure (MAP) and heart rate (HR). The right femoral vein was cannulated for drug administration and hypertonic saline infusion (HSI) (3 M NaCl; Sigma-Aldrich, St. Louis, MO, EUA [10, 25]). The right carotid was cannulated for blood withdrawal. A tracheostomy was performed to reduce airway resistance prior to the placement (in the prone position) of the animals in a stereotaxic apparatus (David Kopf Instruments, Tujunga, CA, USA). A miniature probe (probe 1RB, Transonic Systems, Inc., Ithaca, NY, USA) was implanted around the left renal artery for renal blood flow (RBF) recording while the second miniature probe (probe 2RB, Transonic Systems, Inc., Ithaca, NY, USA) was implanted around the aorta to record aortic blood flow (ABF) as detailed in the previous work [13, 19, 26]. The body temperature of animals was maintained between 36 and 37°C using a heating pad throughout the experiment.

### 2.3. Hemorrhagic Shock

Hypovolemic hemorrhage was induced by slow blood withdrawal over 20 min until the value of MAP reached 60 mmHg. The average volume of 13.3 mL was withdrawn per kg of body weight (13.3 mL/kg).

### 2.4. Nanoinjections in the MnPO

The nanoinjections into the coordinates 0.4 mm rostral to bregma, 0.0 mm lateral to midline, and 7.2 mm below dura mater were performed after hemorrhage induction by using a glass micropipette. In order to block MnPO pharmacologically, muscimol (4 mM) was nanoinjected (100 nL) in experimental group (EXP). Animals from control group (CONT) received saline (150 mM NaCl). For histological confirmation of the nanoinjection site, equal volume of Evans blue solution (4%; Sigma-Aldrich, St. Louis, MO, USA) was nanoinjected, at the end of the experiments, into the same coordinates of previous nanoinjections.

### 2.5. Sodium Overload

The sodium overload through the intravenous infusion of hypertonic saline (his) (3 M NaCl) was realized after 2 min of MnPO nanoinjection. The HSI was performed through a cannula that was implanted in the right femoral vein. The administration was performed within 60 seconds at a dose of 1.8 mL/kg of body weight.

### 2.6. Hematocrit, Plasma Sodium Concentration, and Osmolality

Blood samples (0.2 mL) were collected during the baseline period, 20 min after hemorrhage and 30 and 60 min after HSI. Following each sampling, an equal volume of 0.15 M NaCl was injected to reduce changes in extracellular fluid volume. Part of the sample was used to verify the hematocrit through a glass capillary tube (Perfecta, Cod. 100). The sodium concentration was determined by flame photometer (model 910M, Analyser, São Paulo, SP, Brazil). In order to determine osmolarity, blood samples were centrifuged at 3500 rpm for 5 minutes prior to the separation and analysis of plasma using an osmometer (Osmette II, model no. 5005; Precision Systems Inc., Natick, MA, USA).

### 2.7. Recording of MAP, HR, RBF, ABF, RVC, and AVC

The pulsatile arterial pressure (PAP) of anesthetized animals was continuously recorded through the arterial cannula that was connected to a pressure transducer (MLT0380, ADInstruments, Bella Vista, Australia) with an amplifier (Bridge Amp, ML221, ADInstruments, Bella Vista, Australia). Data were digitized at a frequency of 1000 samples per second using an analogue to digital converter (PowerLab 4/25, ML845, ADInstruments, Bella Vista, Australia). MAP was calculated from the integral of PAP's signal (PowerLab 4/25, ML845, ADInstruments, Bella Vista, Australia). HR was calculated as instantaneous frequency from the PAP's signal (PowerLab 4/25, ML845, ADInstruments, Bella Vista, Australia).

The miniatures probes were connected to T206 flowmeter (Transonic Systems, Inc., Ithaca, NY, USA), in order to record the RBF and ABF. The signals obtained were recorded by the acquisition and data analysis MP150 system (PowerLab 4/25, ML845, ADInstruments, Bella Vista, Australia). Data were digitized at a sampling frequency of 200 samples per second. Changes in RBF and ABF were calculated as the percentage relative ratio to baseline (%RBF and %ABF).

The RVC and AVC were obtained by the ratio of RBF/MAP and ABF/MAP, respectively. The variations of RVC and AVC were expressed as percentage change in baseline value (%RVC and %AVC, resp.).

### 2.8. Histology

The anesthetized rats were perfused with saline (0.15 M NaCl) and formaldehyde (10%) prior to the removal, postfixation, cryoprotection, and cutting of brains in coronal sections of 40 *μ*m thick with freezing microtome (Leica, Wetzlar, Germany). In order to determine the sites of nanoinjection in MnPO, the slices obtained in this hypothalamic region were stained with neutral red. The stained slices were mounted on slides to localize and take a photograph of MnPO.

### 2.9. Statistical Analysis

Data were expressed as means ± S.E.M. Cardiovascular baseline values were analyzed by unpaired student *t*-test. The effects of HSI on cardiovascular parameters were analyzed using two-way ANOVA followed by Newman-Keuls as post hoc test for detecting pairwise differences. A value of *P* < 0.05 was considered to denote a significant difference.

## 3. Results

### 3.1. Effects of Blockade of MnPO on Cardiovascular Responses to HSI in Rats with Hypovolemic Hemorrhage

**Figure 1:**
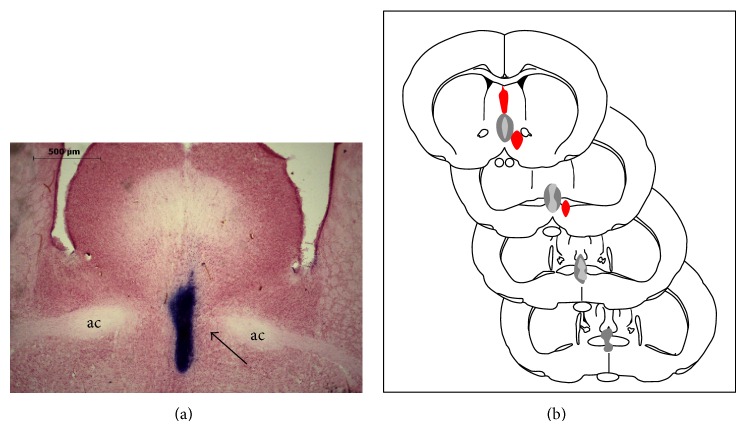
Typical injection site in the median preoptic nucleus (MnPO). (a) Photomicrograph of a coronal section of the forebrain of a representative site following a nanoinjection labeled with 4% Evans blue dye (arrow). (b) Four sequential coronal drawings showing the maximum (dark area) and minimum (gray area) extent of dye diffusion into the MnPO. Red spots indicated the extent of dye diffusion outside of the MnPO.

Analysis of the spread of dye that was microinjected at the end of the experiment showed that the drug injections were confined to the region of MnPO (Figure 1). Only the rats which showed the confinement of Evans blue dye to the MnPO were considered for analysis.

The baseline values of mean arterial pressure (MAP), heart rate (HR), renal blood flow (RBF), aortic blood flow (ABF), renal vascular conductance (RVC), and aortic vascular conductance (AVC) were similar in control (*n* = 6) and EXP (*n* = 6) groups (Table 1).

**Figure 2:**
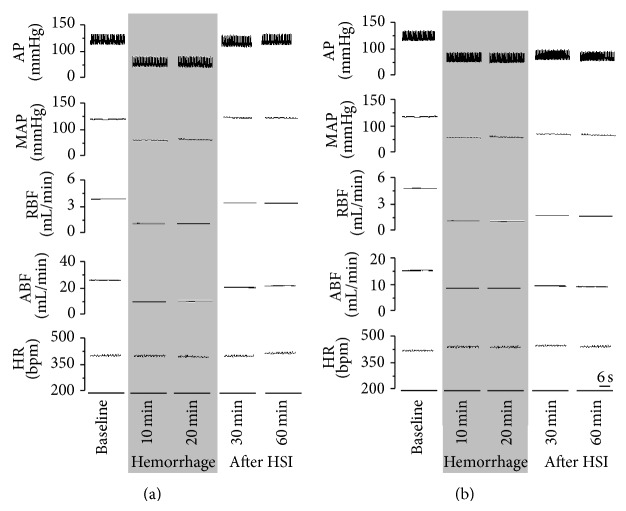
Digitized record of cardiovascular responses to HSI in control (a) and experimental (b) groups. Pulsatile arterial pressure (PAP), mean arterial pressure (MAP), renal blood flow (RBF), aortic blood flow (ABF), and heart rate (HR).

**Figure 3:**
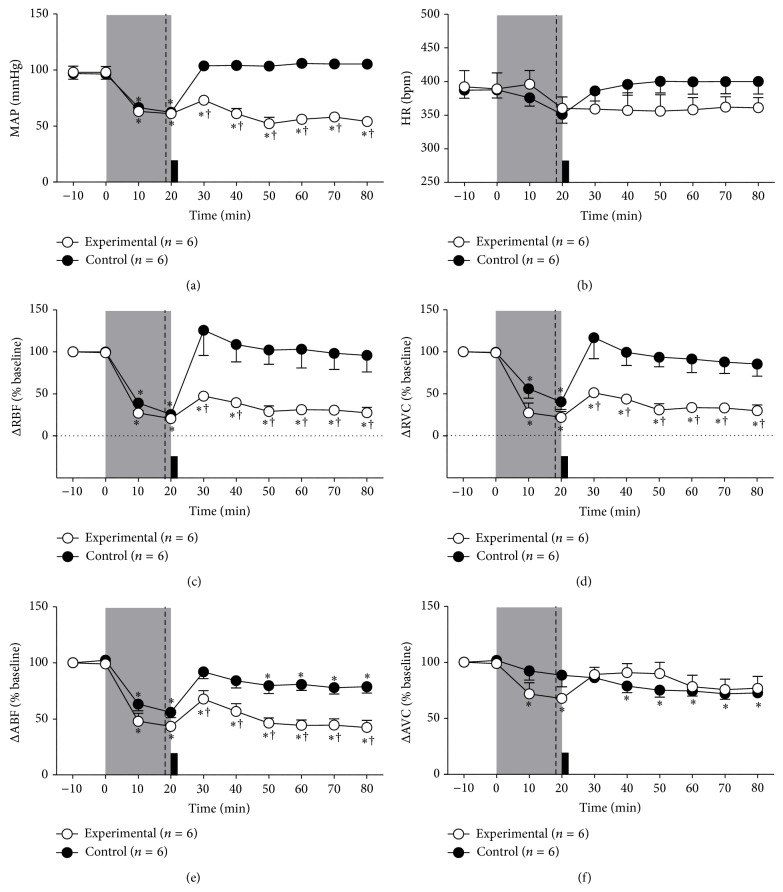
Effects of pharmacological blockade of the MnPO on changes in mean arterial pressure (MAP; (a)), heart rate (HR; (b)), variations of renal blood flow (ΔRBF, % baseline; (c)), renal vascular conductance (ΔRVC % baseline; (d)), aortic blood flow (ΔABF, % baseline; (e)), and aortic vascular conductance (ΔAVC, % baseline; (f)) induced by sodium overload in the rats submitted to hemorrhage. Control group received nanoinjections of 150 mM NaCl (*n* = 6) and experimental group received nanoinjections of 4 mM Muscimol (*n* = 6) groups. Values were expressed as means ± S.E.M.  ^*^Different from time 0; ^†^different from control group with *P* < 0.05. Shaded area indicates the period of hemorrhage. Dashed line represents the moment of nanoinjection into the MnPO (muscimol or vehicle) and black block indicates intravenous hypertonic saline infusion.

Volume of blood that was withdrawn during hemorrhagic shock was similar in both groups (CONT: 3.5 ± 0.5 versus EXP: 3.9 ± 0.4 mL). Hemorrhagic shock elicited equipotent hypotension in CONT (96.9 ± 5.1 to 62.2 ± 1.1 mmHg, after 20 min of hemorrhage, Figures 2(a) and 3(a)) and EXP groups (98 ± 5.4 to 61 ± 0.4 mmHg, after 20 min of hemorrhage, *P* < 0.05, Figures 2(b) and 3(a)). As expected, 10 minutes after HSI, the CONT group showed a complete recovery of hypotension (104 ± 4.2 mmHg). In contrary, MnPO inhibition completely blocked the HSI induced arterial blood pressure recovery (60 ± 6.2 mmHg 10 min after HSI, Figure 3(a)). No significant differences were found in heart rate after hemorrhage induced hypotension (CONT: 351 ± 13.0 versus EXP: 360 ± 17.1 bpm) and at 60 min after HSI (CONT: 400 ± 18.4 versus EXP: 361 ± 15.3 bpm; Figure 3(b)).

There was no difference in the reduction of RBF induced by hemorrhagic shock (CONT: −74 ± 5.4% versus EXP: −78 ± 6.9%; *P* < 0.05; Figure 3(c)). In CONT group, the RBF was restored to baseline value after 10 min of sodium overload (26 ± 3% from baseline at 10 minutes after infusion) and remained in this level throughout the experiment (−4 ± 19.8% over baseline, 60 minutes after the infusion, Figure 3(c)). In EXP group, the RBF was not restored (−70 ± 7.1% at baseline to 60 minutes after infusion, *P* < 0.05; Figure 3(c)).

Hemorrhagic shock induction caused a substantial renal vasoconstriction in both control (−59 ± 9.2% from baseline, 20 minutes after hemorrhage; Figure 3(d)) and EXP groups (−65 ± 10.9% of baseline, 20 minutes after hemorrhage; Figure 3(d)). In addition, CONT group showed restoration and maintenance of RVC to baseline levels after HSI till the end of recordings (−15 ± 14.2% compared to baseline 60 min after infusion; Figure 3(d)). MnPO blockade in EXP group maintained the renal vasoconstriction after hemorrhage. This response caused by MnPO inhibition was maintained throughout the experimental period (−48 ± 9.7% compared to baseline value after 60 min of infusion, *P* < 0.05; Figure 3(d)).

Both groups of animal showed equipotent decrease in ABF after hemorrhage (CONT: −44 ± 4.8% versus EXP: −56 ± 4.0% compared to 0 minutes). HSI, in CONT group, induced a rapid ABF restoration to baseline (−8 ± 6.0% after 20 min of infusion as compared to 0 minutes), and a subsequent decrease at the end of experiments (−21 ± 5.6% after 60 min of infusion as compared to 0 minutes; Figure 3(e)). In EXP group, the sodium overload was not able to restore ABF (−58 ± 6.2% after 60 min of infusion as compared to baseline, *P* < 0.05, Figure 3(c)).

Regarding AVC, no significant difference was observed (CONT: −27 ± 4.3% versus EXP: −23 ± 10.6% as compared to baseline). However, EXP group animals maintained their conductance levels similar to baseline while a mild aortic vasoconstriction was observed in control group (CONT: −21 ± 5.9% versus EXP: −9 ± 8.1% as compared to baseline after 20 min of infusion, *P* < 0.05, Figure 3(f)).

When muscimol was nanoinjected into regions adjacent to, but outside of, the MnPO (Figure 1; red spots; *n* = 3), we observed that HSI promotes similar responses to control rats: recovery of MAP (101 ± 7.2 mmHg after 50 min of infusion as compared to 0 minutes), HR (386 ± 1.8 bpm after 50 min of infusion as compared to 0 minutes), RBF (100 ± 10% as compared to baseline after 50 min of infusion as compared to 0 minutes), RVC (98 ± 2.1% as compared to baseline after 50 min of infusion as compared to 0 minutes), ABF (99 ± 7.4% as compared to baseline after 50 min of infusion as compared to 0 minutes), and AVC (98 ± 1.7% as compared to baseline after 50 min of infusion as compared to 0 minutes), by HSI after hemorrhagic shock induction, showing that nanoinjections of muscimol outside the MnPO did not abolish cardiovascular recovery by HSI in hemorrhagic-rats.

### 3.2. HSI Effects on Hematocrit, Plasma Sodium, and Osmolarity

**Figure 4:**
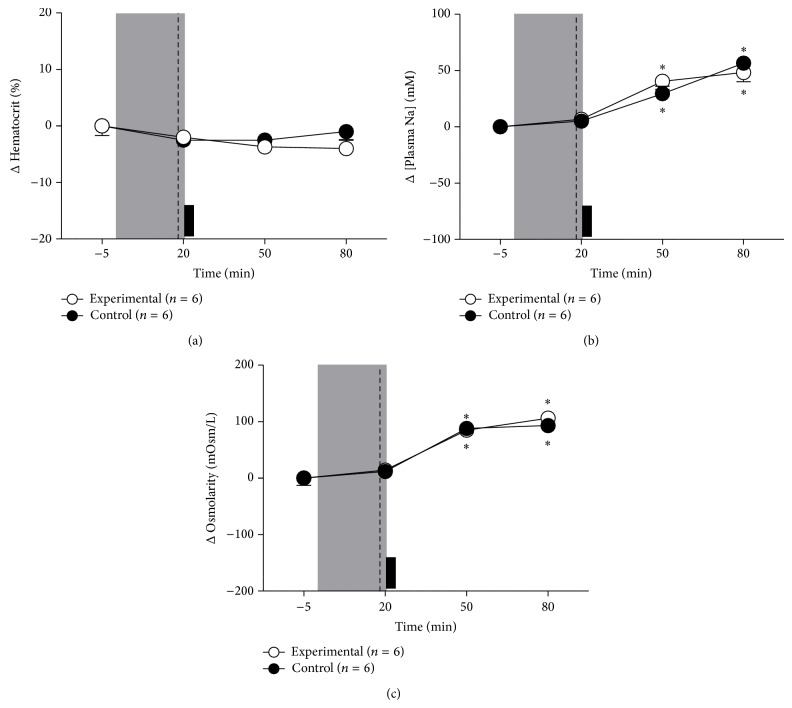
Effects of pharmacological blockade of the MnPO on changes in hematocrit level (a), plasma sodium concentration (b), and osmolarity (c) induced by hypertonic saline infusion in rats submitted to hemorrhage. Control group received nanoinjections of 150 mM NaCl (*n* = 6) and experimental group received nanoinjections of 4 mM Muscimol (*n* = 6) groups.  ^*^Different from baseline, *P* < 0.05. The shaded area indicates the period of hemorrhage. Dashed line represents the moment of nanoinjection into the MnPO (muscimol or vehicle) and the black block indicates intravenous hypertonic saline infusion.

In control rats, HSI did not change hematocrit (Δ  −1 ± 1.5% as compared to baseline after 60 min of HSI; Figure 4(a)) but increased plasma [Na^+^] (Δ  48 ± 8.1 mM as compared to baseline after 60 min of HSI; Figure 4(b); *P* < 0.05). HSI increased osmolarity (Δ  106 ± 9.3 mOsm/L as compared to baseline after 60 min of HSI; Figure 4(c); *P* < 0.05). In rats submitted to pharmacological blockade of MnPO, hypertonic saline HSI did not change hematocrit (Δ  −4 ± 1.6% as compared to baseline after 60 min of HSI; Figure 4(a)); however, it increased plasma [Na^+^] (Δ  56 ± 5.5 mM as compared to baseline after 60 min of HSI; Figure 4(b)) and osmolarity (Δ  93 ± 7.2 mOsm/L as compared to baseline after 60 min of HSI; Figure 4(c)). These results were similar with those observed in control group.

## 4. Discussion

Several experimental evidences suggest that MnPO is important for maintenance of the composition and volume of the extracellular compartment. The MnPO receives inputs from the circumventricular organs and peripheral osmoreceptors with reciprocal connections with paraventricular nucleus of hypothalamus (PVN), parabrachial nucleus, and ventrolateral medulla that are involved in cardiovascular regulations [14, 27–31]. Although previous studies have demonstrated that the MnPO plays an important role in hormonal, autonomic, and cardiovascular responses induced by changes in osmolality and circulating volume in normovolemic rats [16–19, 21, 25, 32], the involvement of MnPO in the HSI mediated cardiovascular recovery during hypovolemic hemorrhage remains unknown. The present study provides new key observations such as prevention.

In the last decades, several studies have shown that hyperosmolarity induced by HSI brings great benefits to the hypotension caused by shock. This shock could lead to the development of complications, multiple organ failure, and susceptibility to infections such as pneumonia and sepsis or result in the death of victim [3, 4]. HSI induces a prompt recovery of MAP, cardiac output reestablishment and restoration of mesenteric, and renal and hindlimb circulation. This cardiovascular recovery depends on the activation of sympathetic nervous activity and angiotensinergic and vasopressinergic mechanisms [7, 8, 33–36]. In agreement with the findings of these pioneer studies, our results showed that hypertonic saline is effective in restoring the cardiovascular parameters of hypovolemic animals.

Hypernatremia activate various neuronal reflex mechanisms for homeostasis restoration in severe hemorrhagic cases [9–11, 33, 37]. Some authors demonstrated that sinoaortic denervation abolishes the renal vasodilation induced by hypernatremia unlike bilateral vagotomy (removal of cardiopulmonary receptors) that does not alter this response [38, 39]. These results suggest that fibers associated with aortic and carotid baroreceptors play an important role in detecting variations in pressure and/or blood volume, which may be involved in detecting extracellular fluid composition. Corroborating these data, other findings showed that the combined removal of baroreceptors and chemoreceptors increases hypotension induced by hemorrhage, thus confirming that these interceptive receptors are important to adjust blood pressure during hypovolemia [40, 41].

Younes et al. [36] and Lopes et al. [33] demonstrated that a section vagus nerve blockade or denervation of one lung abolishes the beneficial cardiovascular effects of hypertonic saline in dogs submitted to hemorrhage. The authors observed that the reversible blockade of vagal activity abolished the compensatory mechanisms. These results show that the hypertonic resuscitation depends not only on direct effect of hyperosmolarity on vascular reactivity and myocardial contractility but also on a neural component.

The anteroventral wall of third ventricle (AV3V) that includes the organum vasculosum lamina terminalis (OVLT), the ventral portion of the MnPO, the preoptic periventricular nucleus, and the more medial aspects of the medial preoptic nucleus is an important region that is involved in body fluid and cardiovascular regulation [25, 32, 37]. The study of Barbosa et al., [37] showed that electrolytic lesion of AV3V impaired the recovery of arterial pressure induced by HSI in hemorrhagic rats. However, it is important to emphasize that the electrolytic ablation of the AV3V promotes unspecific lesion of different nuclei and crossing fibers. Our study is more specific through pharmacological inhibition of the MnPO to impair the HSI induced resuscitation in hemorrhagic rats. Additionally, we can clarify that MnPO is the mediator of hyperosmotic recovery mechanisms in hemorrhagic shock. Meanwhile, to the best of our knowledge, no other study has demonstrated that MnPO activation is essential for the HSI induced cardiovascular recovery during hypovolemic shock.

Recently we have demonstrated that the pharmacological blockade of the MnPO by muscimol causes reduction of 5 mmHg of MAP during just 40 s [26]. So, it is not possible to assume that the nonrecovery of cardiovascular parameters in rats that were subjected to blockade of the MnPO is due to a prolonged drop in MAP induced by muscimol nanoinjection into this nucleus. In addition, hemorrhagic rats that received nanoinjection of muscimol in areas close to MnPO have the same recovery of MAP induced by HSI. These results demonstrated that the specific inhibition of MnPO abolished HSI induced resuscitation in hemorrhagic rats.

Evidences have been established that changes in plasma osmolarity were detected by central structures, such as the organum vasculosum of the lamina terminalis (OVLT) and subfornical organ (SFO), and sent through excitatory projection to MnPO [42]. The MnPO also receives projections from catecholaminergic A2 neurons present in NTS (an area of the CNS that integrates afferences from the carotid and peripheral osmoreceptors) [14, 15, 43]. Current data show that HSI did not reverse the hemorrhage in animals that were subjected to pharmacological blockade of GABA_A_ receptors in MnPO since sodium overload did not restore blood pressure. Moreover, the standard response to HSI (renal vasodilation) was abolished in animals that were submitted to MnPO blockade. The results of this study demonstrated that projections from MnPO constitute the pathways that control changes in circulating blood volume, carotid sensors, and central osmoreceptors.

The main central osmoreceptors (OVLT and SFO) and MnPO that are located in the forebrain lamina terminalis [9, 32, 42, 44] target PVN primarily. In fact, neuroanatomical and electrophysiological studies have demonstrated projections from the MnPO to neurons of the PVN [21, 45, 46]. The PVN is established as a leading hypothalamic center that controls the cardiovascular system and is involved in the regulation of body fluids [47–49]. The neurons of this nucleus modulate sympathetic nervous activity through projections to rostral ventrolateral medulla and intermediolateral column of spine cord and vasopressin secretion [44, 50–52]. It has been shown that a hyperosmotic challenge recruits the MnPO neurons that project to the PVN [21], demonstrating that the MnPO-PVN pathway controls the response to the sensitization of central osmoreceptors. The integrity of this pathway has great importance in the modulation of autonomic reflex adjustments in HSI induced recovery during bleeding. Therefore, the MnPO could be assumed to be an integrative center of a complex central pathway responsible for cardiovascular recovery induced by HSI during hemorrhagic shock mainly through sympathetic modulation and vasopressin release.

## 5. Conclusion

The results in this study suggest that the activity of neurons in the MnPO is critical to the restoration of blood pressure and renal perfusion induced by infusion of hypertonic saline in animals that are subjected to hemorrhagic shock.

## Figures and Tables

**Table 1:** Baseline values of mean arterial pressure (MAP), heart rate (HR), renal blood flow (RBF), aortic blood flow (ABF), renal vascular conductance (RVC), and aortic vascular conductance (AVC) in the control (CONT) and experimental (EXP) groups.

Group	*N*	MAP (mmHg)	HR (bpm)	RBF (mL/min)	ABF (mL/min)	RVC mL/(min∗mmHg)	AVC mL/(min∗mmHg)
CONT	6	97 ± 5.1	387 ± 12.0	2.8 ± 0.3	17.8 ± 2.4	0.02 ± 0.004	0.15 ± 0.017
EXP	6	98 ± 5.4	392 ± 24.1	3.6 ± 0.7	19.1 ± 2.8	0.03 ± 0.006	0.16 ± 0.023

Values were expressed as means ± S.E.M.
